# New insights on the phytochemical intervention for the treatment of neuropsychiatric disorders using the leaves of *Michelia champaca*: an *in vivo* and *in silico* approach

**DOI:** 10.1080/13880209.2022.2101669

**Published:** 2022-09-02

**Authors:** Pushpa V. H., Jayanthi M. K., Rashmi H. R., Veeresh Kumar N. Shivamurthy, Shashank M. Patil, Prithvi S. Shirahatti, Ramith Ramu

**Affiliations:** a Department of Pharmacology, JSS Medical College, JSS Academy of Higher Education and Research, Mysuru, India; b Department of Neurology, Saint Francis Hospital and Medical Center, Trinity Health Of New England, Hartford, CT, USA; c Department of Biotechnology and Bioinformatics, JSS Academy of Higher Education and Research, Mysuru, India; d Department of Biotechnology, Teresian College, Mysuru, India; e St. Joseph's College for Women, Mysore, Karnataka, India

**Keywords:** Antidepressant activity, anxiolytic activity, Magnoliaceae family, forced swim test, tail suspension test, molecular docking studies

## Abstract

**Context:**

*Michelia champaca* L. (Magnoliaceae) has been known since ancient times for its rich medicinal properties.

**Objective:**

The ethanol extract of *Michelia champaca* leaves (EEMC) was evaluated on depression and anxiety using *in vivo* and *in silico* studies

**Materials and methods:**

Swiss albino mice were divided into control, standard, 100 and 200 mg/kg b.w. EEMC groups and for drug administration using oral gavage. The antidepressant activity was evaluated using forced swim test (FST) and tail suspension test (TST) whereas the anxiolytic activity through elevated plus maze and light and dark tests. The *in silico* studies included molecular docking against human potassium channel KCSA-FAB and human serotonin transporter, and ADME/T analysis.

**Results:**

Open arm duration and entries were comparable between 200 mg/kg b.w. group (184.45 ± 1.00 s and 6.25 ± 1.11, respectively) and that of diazepam treated group (180.02 s ± 0.40 and 6.10 ± 0.05, respectively). Time spent in the light cubicle was higher (46.86 ± 0.03%), similar to that of diazepam (44.33 ± 0.64%), suggesting its potent anxiolytic activity. A delayed onset of immobility and lowered immobility time was seen at both the treatment doses (FST: 93.7 ± 1.70 and 89.1 ± 0.40 s; TST: 35.05 ± 2.75 and 38.50 ± 4.10 s) and the standard drug imipramine (FST: 72.7 ± 3.72 and TST: 30.01 ± 2.99 s), indicative of its antidepressant ability. *In silico* studies predicted doripenem to induce anxiolytic and antidepressant activity by inhibiting human potassium channel KCSA-FAB and human serotonin transporter proteins, respectively.

**Conclusions:**

EEMC is a rich source of bioactive compounds with strong antidepressant and anxiolytic properties.

## Introduction

Depression is a condition caused by a persistent sadness and lack of interest to carry out routine activities. A person is considered clinically depressed if he shows a persistent feeling of sadness and lack of interest for at least a period of 2 weeks or more. Lower mood, difficulties in thinking, loss of interest, and disruption in the normal routine such as disrupted sleep, loss of energy, and change in sex desire are all common symptoms of depression (Porter and Meldrum 2009). Characteristics such as loss of appetite, weight, sleep disturbances, psychomotor activity, decreased energy, feeling of worthlessness, guilty and suicidal ideation are commonly seen (Fauci et al. 2008). Major depression is estimated to be around 5% among the general population while a female to male ratio prevails at 5:2. The World Health Organization (WHO) reported that about 450 million people exhibited certain amount of mental or behavioural disorder, which is often left undetected or untreated with the fear of the societal circumstances (Tsegay et al. 2020).

Anxiety is also a mental condition leading to a state of extreme trepidation and uncertainty as a result of anticipation of a future threat. When anxiety and depression coexist, it predicts poor outcomes and a higher rate of treatment resistance than when the two disorders occur separately. The presence of anxiety and depression together complicates diagnosis and makes treatment difficult. Individuals suffering from coronary heart diseases, diabetes and stroke have an increased risk of developing anxiety and depression at some point in their life. In addition, there is an increased risk of suicide rate among the depressed patients (Bhatt et al. 2020). Drugs that target the levels of monoamines, namely, noradrenaline, dopamine and serotonin are generally used to treat this condition (Reddy 2010). However, their success rate is only up to 60% and requires prolonged administration for visible improvement in the signs and symptoms. They are always associated with numerous side effects owing to their effects on the brain and neurotransmission (Kumar et al. 2004; Jawaid et al. 2011). Advancements in research have identified some of the newer drugs that are associated with fewer side effects. However, fewer than half of those affected in the world do not receive such treatments mainly due to barriers such as lack of resources, lack of trained health-care providers, and social stigma associated with mental disorders. Therefore, it becomes highly necessary to look for naturally occurring alternatives from plant sources with proven advantage and favourable benefit to risk ratio.

In addition, major depressive disorder is caused by one or more of the following namely, genetic predisposition, unregulated monoamine synthesis and regulation as well as altered brain structure (Hasler 2010). Even the environmental and socioeconomic factors contribute significantly to the development of clinical depression. Studies that have incorporated targeted treatment for the regulation of monoamine production and functions (such as tricyclic antidepressants, serotonin reuptake inhibitors) have fared better in the treatment of depression, suggesting its pivotal role in the development of depression (Fedoce et al. 2018; Greaney et al. 2019; Bhatt et al. 2020).

In this regard, plants have been a valuable source of natural products for maintaining human health for many years. *Michelia champaca* L. (Magnoliaceae) is a folk-medicinal plant well known for its antioxidant and antidiabetic properties. It has traditionally been used to treat diarrhoea, cough, bronchitis, hypertension, dyspepsia, fever, rheumatism, abscesses, dysmenorrhoea and inflammation. While the dried root and bark of *M. champaca* is used as purgative, expectorant, cardio tonic, digestive, carminative, stimulant, the flower of *M. champaca* is used as diuretic, diaphoretic, antipyretic, the leaves help as a colic aid and the bark is used as an astringent agent. Several studies report its antipyretic, anti-inflammatory antioxidant antimicrobial, cytotoxic, antidiabetic and analgesic activities (Nickavar et al. 2006; Hossain et al. 2009; Taprial 2015). *M. champaca* contains various bioactive constituents including alkaloids, palmitic acid, oleic acid, carbonyl acid and esters, oliveroline, lysicamine, nornuciferine, cyperone, ficaprenolepi-yangambin, pheophytin, aristophyll, michephyll and formylanonaine, which is likely the reason for these innumerable medicinal properties (Hoffmann et al. 1977; Khan et al. 2002; Jarald et al. 2008; Wang et al. 2010; Chandra et al. 2015).

Disease conditions such as inflammation bring about oxidative stress, which is characterized by an imbalance in the regulation of reactive oxygen species (ROS) and inability of the body to detoxify the generated redox intermediates. Plant-based medicines have an added advantage mainly by countering these ROS productions and in turn reduce inflammation caused by their action (Halliwell and Whiteman 2004; Patil, Shirahatti, et al. 2021). Various studies have reported the use of root, bark and flowers of *Michelia champaca* for other activities (Swantara et al. 2020). However, the perusal of literature reveals that, so far, no attempt is made to study the role of *M. champaca* leaves in relation to its anxiolytic and anti-depressant properties and therefore an attempt was made to elucidate its effects in depression and anxiety.

## Materials and methods

### Animal model

Swiss albino mice of either sex weighing 25–30 kg were procured from the Central Animal House, JSS Medical College, Mysuru, India. The animals were maintained under ambient temperature (25 °C) and humidity (45–55%). All the animals were given free access to food and water with a natural day and night cycle. The experiments were carried out after obtaining clearance from the Institutional Animal Ethics Committee of JSS Medical College, Mysuru, Karnataka, India dated 18 March 2019, proposal no. 260-2017. Table 1 provides a detailed description of the various inclusion and exclusion criteria to employ animals for the experiment.

**Table 1.** Criteria for the selection of animals for the study.

Inclusion criteria	Exclusion criteria
Healthy Swiss Albino mice of either sex Weight: 25–30 g Age: 3–4 months Healthy mice with normal behaviour and activity.	Unhealthy animal Obese animal Pregnant animal Animals previously used in other experiments

### Chemicals

Imipramine, diazepam (Sisco Research Laboratories, Mumbai, India) were used as a standard drug for antidepressant and anxiolytic activity. Saline and ethanol Merck (Mumbai, India) were used for plant material preparation.

### Instruments

Glass cylinder (25 × 12 × 25 cm), metal lever and stop watch, oral gavage, glass tubes, sterile tuberculin and insulin syringes, glass beakers, cotton and glass rod stirrer.

### Plant material and preparation of drug solution

Fresh leaves of *M. champaca* were collected in the month of October 2018 from Honnuru village, Mysore, Karnataka, India. The leaves were authenticated by the taxonomist (Prof. Siddaramaiah) working at the Department of Horticulture, Government of Karnataka, Mysore, India and a voucher specimen was submitted in the herbarium at the same department with a specimen number 66786 MYS. The leaves that were initially washed with 70% alcohol were then shade-dried and powdered coarsely. Further, about 500 g of this powder was mixed with ethanol and subjected to Soxhlet extraction under room temperature for a period of 24 h. This was further subjected to vacuum evaporation for the removal of the solvent. The concentrated ethanol extract of the leaves of *Michelia champaca* (EEMC) was used for further experiments to evaluate the antidepressant activity. For the study, a stock solution was prepared by dissolving EEMC in saline, which was then adjusted to various concentrations using saline. The experimental procedure was carried out with two dosing schedules.

### High-resolution liquid chromatography and mass spectrometry analysis

EEMC was analysed using high-resolution liquid chromatography and mass spectrometry (HR-LCMS) at the advanced analytical instrument facility (SAIF), IIT-Bombay, Mumbai, India. Chemical fingerprints of the selected medicinal plant extracts were prepared by Agilent HR-LCMS model-G6550A with 0.01% mass resolution. The acquisition method was set to be MS – minimum range 150 (*m/z*) and maximum 1000 Da (*m/z*), with the scanning rate each spectrum per second. Liquid chromatography was maintained at 40 °C, with HIP sampler (1000 µL/min) and binary sampler (0.300 mL/min). Chromatographic separations were performed on a column (100 mm × 1.0 mm, particle size 1.8 μm; Waters, Milford, MA), where 10 μL of sample was injected with flush out factor 5.0 s. The solvent system used for HR-LCMS was 100% water in pump A, and 100% acetonitrile in pump B. The SAIF (IIT-Bombay, Mumbai, India) database, which has over 62,000 patterns was used to identify components in the extract and analyse them using mass spectrometry HR-LCMS. The unknown component's spectrum was compared with that of the known components recorded in the SAIF library.

### Acute toxicity evaluation

The study animals were taken in two phases. In the first phase, nine mice divided into three groups were administered doses of 10, 100 and 1000 mg/kg of EEMC and monitored for seven days in order to evaluate acute toxicity. Further, in the second phase, three animals were treated with 1200, 1500 and 2000 mg/kg body weight and monitored for a week to check for behavioural changes, if any.

## Experimental design

A total of 48 mice were used for this study and divided into four different groups for each experimental model. Each group consisted of six animals.

### Antidepressant activity (24 mice)

*Group I*: Control treated with normal saline (10 mL/kg).

*Group II*: Standard drug for antidepressant activity – imipramine (15 mg/kg).

*Group III*: Ethanol extract *M. champaca* leaves (EEMC-100 mg/kg).

*Group IV*: Ethanol extract *M. champaca* leaves (EEMC-200 mg/kg).

### Anxiolytic activity (24 mice)

*Group V*: Control treated with saline.

*Group VI*: Standard drug for anxiolytic activity – diazepam (5 mg/kg, b.w.; i.p.).

*Group VII*: Ethanol extract *M. champaca* leaves (EEMC-100 mg/kg).

*Group VIII*: Ethanol extract *M. champaca* leaves (EEMC-200 mg/kg).

All the groups were treated with vehicle, test drug EEMC (100 and 200 mg/kg) and standard drug for a period of seven days. On the 7th day, after one hour of drug treatment, animals were screened for antidepressant and anxiolytic activities.

### Anxiolytic activity

#### Elevated plus maze test

This is one of the widely accepted tests to assess anti-anxiety properties of drug molecules in rodent models as well as to assess the underlying mechanism (Walf and Frye 2007). The study was performed on 24 mice taken in four different groups of six animals each as stated previously. The study design consisted of two open arms (50 cm long and 10 cm wide) and two closed arms (50 cm long, 10 cm wide and 38 cm height) that were placed opposite to each other in a 10 × 10 platform. The animals were exposed to the maze for 5 min, which was followed by recording of the number of entries in open and closed arms and the duration spent in both the arms (Riaz and Khan 2014).

#### Light and dark test

The light and dark test was performed as per the method given by Gong et al. (2006). With a span of 30 min between the tests, the same animals used in the above assay were subjected to the light and dark test. The study design comprised of a dark safe cubicle and an aversive light cubicle with a dimension of 1/3 for dark and 2/3 for light compartment and an external size of 46 × 27 × 30 cm. The mice were initially placed at the midpoint of the light cubicle facing towards the light cubicle. The time each animal spends was recorded for 5 min and then returned to their home cage by holding them with their tail. Further, the maze was sanitized using 10% ethanol and dried between tests. Anxiety was recorded as high if the mice spent less than 50% of time in the light compartment.

*Acute study*: The drugs were administered as a single dose 1 h prior to the observation.

*Chronic study*: The drugs were administered for seven days continuously. On the 8th day morning, outcome observations were obtained.

#### Antidepressant activity

The antidepressant activity was evaluated using experimental models of depression namely, (i) forced swim test (FST) and (ii) tail suspension test (TST). Animals were weighed and appropriate dose of the drug was administered orally to different groups.

#### Forced swim test

The FST was performed according to the procedure given by Porsolt et al. (1977). This is a standard test for screening of the antidepressant activity. Briefly, a cylindrical container filled with water 20 cm depth was taken and the experimental mice were forced to swim in this water for a period of 6 min. Once the mice stopped its struggle and floated on water, it was considered immovable, which was taken as the end point in the study. The duration of immobility was assayed during the last 4 min after treatment.

#### Tail suspension test

The TST was performed according to the method of Steru et al. (1985). Mice were trapped 1 cm from its tail such that they were suspended 75 cm above the ground. This treatment was performed for 6 min and the mice were considered immovable when it refrained from demonstrating escape-oriented behaviour. The total immobility duration was assayed for the last 4 min.

## *In silico* studies

### Molecular docking and virtual screening

Crystallographic 3D structures of human potassium channel KCSA-FAB (PDB ID: 4UUJ) and human serotonin transporter (PDB ID: 516X) proteins were retrieved from Protein Data Bank RCSB PDB (https://www.rcsb.org/). Autodock Tools 1.5.6 was used for protein and ligand preparation according to the Autodock 4.0 methodology which uses Lamarckian Genetic Algorithm (LGA) (Patil, Martiz, Ramu, Shirahatti, Prakash, Chandra, et al. 2021). Proteins were cleaned by removing water and ions. Polar hydrogens were added and non-polar hydrogens were merged into their corresponding carbons for the appropriate treatment of electrostatics, subsequently leading to the protein stabilization. The protein structures were further processed with energy minimization by adding Kollman united and Gasteiger charges, prior to assigning AD4 atom type for all the atoms of the protein structures. Alternatively, 3D chemical structures of the 69 phytochemical compounds of *M. champaca* were obtained from PubChem database (https://pubchem.ncbi.nlm.nih.gov/). For docking simulation with human potassium channel KCSA-FAB, diazepam was used as the reference ligand. Alternatively, imipramine was used in case of human serotonin transporter. Ligand preparation was done using Autodock Tools 1.5.6, where the energy minimization was done by applying Kollman united and Gasteiger charges. The torsion of the ligands was set to default, as predicted by the Autodock Tools 1.5.6 software. For the conversion of protein and ligand file formats, OpenBabel 2.3.1 (Patil, Martiz, Ramu, Shirahatti, Prakash, Kumar, et al. 2021) software was used.

Binding pockets of the proteins were positioned in their respective grid boxes. For human potassium channel KCSA-FAB, the grid box of the size 42 Å×42 Å×42 Å was prepared, and was centred at the coordinates *x* = 33.953646, *y* = −25.173089 and *z* = −0.221643. In case of human serotonin transporter, the grid box sized 30 Å×30 Å×30 Å was centred at the coordinates *x* = −32.605542 Å, *y* = −21.385708 Å and *y* = 1.852667 Å. Autodock Vina 1.1.2 was used to dock the ligands to the proteins (Trott and Olson 2010). Ligand molecules were allowed with 10 degrees of freedom. During the docking process, ligands were considered to be flexible, and the protein was assumed to be rigid. Out of the 10 binding poses generated, the first binding pose with no root mean square deviation (RMSD) of atomic positions was considered to be highly valid. They also possess the most negative binding affinity, which indicates stronger binding efficiency. Based on the binding affinity, total number of non-bonding interactions, and respective hydrogen bonds, the extent of protein–ligand interaction was analysed using Biovia Discovery Studio Visualizer 2021 (Kumar et al. 2021).

### Drug-likeness and toxicity analysis

Drug-likeness (a parameter that describes how ‘druglike’ a given substance is) and toxicity factors are essential for evaluating the *in silico* drug pharmacological potential. Any experimental chemical being investigated as a prospective drug candidate should have a high safety margin, as well as an appropriate drug-likeness and toxicological profile. Therefore, using OSIRIS property explorer (http://www.cheminfo.org/Chemistry/Cheminformatics/Property_explorer) (Patil, Maruthi, et al. 2021), the drug-likeness and toxicological properties of the selected phytoconstituents from the previous virtual screening was evaluated. All the structures were submitted to the server in SMILES format.

### Statistical analysis

Statistical comparisons between the normal and treatment groups were performed by one-way analysis of variance (ANOVA), followed by Duncan’s multiple range test using SPSS Software (version 21.0; Chicago, IL). Results were expressed as mean ± SE. The results were considered statistically significant if the *p* values were 0.05 or less.

## Results

### Phytochemical screening test

Freshly prepared EEMC was subjected to phytochemical screening tests for the detection of various active constituents. The extract showed the presence of alkaloids, tannins, steroids, phenols, flavonoids, carbohydrates and glycosides.

Furthermore, the HR-LCMS results revealed the presence of a wide array of compounds that could be indicated with several biological properties. The name and molecular weight of the test materials' components were determined during HR-LCMS (Tables 2a and 2b).

**Table 2a.** Chemical profile of the EEMC of *M. champaca* by high-resolution liquid chromatography and mass spectrometry in + electron spray ionization mode.

S. no.	Name of the compound	Ret. time	Mass	Molecular formula	*m/z*	Difference (ppm)
1	Methyldopa	0.858	211.085	C_10_H_13_NO_4_	212.09	–2.43
2	3-Methyldioxyindole	1.101	163.0632	C_9_H_9_NO_2_	164.07	0.58
3	Lentiginosine	1.282	157.1103	C_8_H_15_NO_2_	158.11	–0.13
4	1-Dehydro-9-fluoro-11-oxotestololactone	3.69	332.1395	C_19_H_21_FO_4_	355.1285	8.81
5	Memantine	6.126	179.17	C_12_H_21_N	202.15	–14.76
6	Betaxolol	7.586	307.215	C_18_H_29_NO_3_	308.22	–0.25
7	6-(1,2,3,4-Tetrahydro-6-methoxy-2-naphthyl)-2(1H)-pyridone	8.281	255.1262	C_16_H_17_NO_2_	256.1335	–1.01
8	1-Phosphatidyl-1D-myoinositol 3-phosphate	8.38	470.0252	C_11_H_20_O_16_P_2_	493.016	–5.44
9	Dihydrodeoxystreptomycin	8.962	567.2897	C_21_H_41_N_7_O_11_	568.2967	–5.82
10	*trans*-1,4-*bis*(2-Chlorobenzaminomethyl)cyclohexane	9.06	390.1604	C_22_H_28_Cl_2_N_2_	391.1674	6.5
11	Beclomethasone	9.07	408.1709	C_22_H_29_ClO_5_	409.1779	–1.27
12	Veraguensin	9.075	372.1939	C_22_H_28_O_5_	373.201	–0.68
13	Momilactone A	9.093	314.191	C_20_H_26_O_3_	337.1803	–8.8
14	3,6-Dimethoxyestra-1,3,5(10),6,8-pentaene-17beta-carboxylic acid methyl ester	9.1	354.1831	C_22_H_26_O_4_	355.1906	–0.05
15	Exemestane	9.106	296.1798	C_20_H_24_O_2_	319.1692	–7.24
16	Longistylin C	9.107	278.1675	C_20_H_22_O	279.1748	–1.57
17	1-(beta-d-Ribofuranosyl)-1,4-dihydronicotinamide	10.117	256.1047	C_11_H_16_N_2_O_5_	279.0939	4.61
18	Carbosulfan	10.374	380.2132	C_20_H_32_N_2_O_3_S	403.2016	0.33
19	Sodium glycocholate	10.943	465.3094	C_26_H_43_NO_6_	466.3165	–0.87
20	Sphinganine	11.545	301.2982	C_18_H_39_NO_2_	302.3056	–0.4
21	Mitoxantrone	11.722	444.2055	C_22_H_28_N_4_O_6_	445.2127	–10.28
22	Butyl 2-aminobenzoate	11.821	193.1108	C_11_H_15_NO_2_	194.118	–2.56
23	Azafrin	12.826	426.278	C_27_H_38_O_4_	427.2842	–2.36
24	Fluticasone propionate	12.967	500.1854	C_25_H_31_F_3_O_5_S	501.1928	–1.87
25	1-Octen-3-yl glucoside	12.989	290.1707	C_14_H_26_O_6_	313.1602	7.85
26	Austinol	12.989	458.1956	C_25_H_30_O_8_	481.1856	–3.44
27	Trenbolone	12.99	270.1628	C_18_H_22_O_2_	293.1539	–2.86
28	Imazamethabenz	12.995	274.1363	C_15_H_18_N_2_O_3_	275.1432	–16.49
29	(*E*,*E*)-1,7-Diphenyl-4,6-heptadien-3-ol	13.001	264.1516	C_19_H_20_O	265.1591	–0.86
30	Panaquinquecol 1	13.143	292.2044	C_18_H_28_O_3_	293.2118	–1.85
31	Kanamycin	13.279	484.2397	C_18_H_36_N_4_O_11_	507.2289	–3.33
32	Dihydrospheroidene/methoxyneurosporene	13.36	570.4661	C_41_H_62_O	308.2223	24.42
33	(9*Z*,11*E*,13*E*,15*Z*)-4-Oxo-9,11,13,15-octadecatetraenoic acid	13.569	290.1887	C_18_H_26_O_3_	291.1959	–1.73
34	Alangimarckine	13.571	475.2846	C_29_H_37_N_3_O_3_	476.2914	–2.28
35	Diplodiatoxin	13.572	308.1994	C_18_H_28_O_4_	309.2066	–2.15
36	3-Phenylpropyl propanoate	13.606	192.115	C_12_H_16_O_2_	193.1228	0.16
37	Deoxytubulosine	13.634	459.2889	C_29_H_37_N_3_O_2_	460.2962	–0.7
38	MG(22:6(4*Z*,7*Z*,10*Z*,13*Z*,16*Z*,1 9*Z*)/0:0/0:0)	14.249	402.2797	C_25_H_38_O_4_	425.2684	–6.7
39	Vulgarone A	14.321	218.1673	C_15_H_22_O	219.1746	–1.26
40	19-Noretiocholanolone	14.526	276.2094	C_18_H_28_O_2_	277.2167	–1.55
41	Hydroxyprogesterone caproate	14.585	428.2933	C_27_H_40_O_4_	429.30	–1.55
42	17-Hydroxylinolenic acid	14.601	294.2198	C_18_H_30_O_3_	295.2271	–1.1
43	Norethindrone enanthate	14.783	410.2822	C_27_H_38_O_3_	411.2893	–0.23
44	Hydroflumethiazide	15.338	330.9958	C_8_H_8_F_3_N_3_O_4_S_2_	353.986	–15.09
45	Lyngbyatoxin	15.396	437.3053	C_27_H_39_N_3_O_2_	460.2943	–2.52
46	Heteratisine	15.403	391.2265	C_22_H_33_NO_5_	392.2334	24.02
47	Linoleoyl ethanolamide	15.622	323.283	C_20_H_37_NO_2_	324.2903	–1.7
48	Pheophorbide b	16.269	606.2477	C_35_H_34_N_4_O_6_	607.2556	0.22
49	7-*O*-Acetylaustroinulin	16.771	364.2616	C_22_H_36_O_4_	365.269	–0.67
50	Virol B	16.846	262.1934	C_17_H_26_O_2_	263.2006	–0.28
51	Harderoporphyrin	17.164	608.2634	C_35_H_36_N_4_O_6_	609.2708	0.17
52	Pheophorbide a	17.925	592.2685	C_35_H_36_N_4_O_5_	593.2759	0.02
53	Pyropheophorbide a	18.492	534.2631	C_33_H_34_N_4_O_3_	535.2701	0.01
54	C16 Sphinganine	10.248	273.2674	C_16_H_35_NO_2_	274.2746	–2.14

**Table 2b.** Chemical profile of the EEMC of *M. champaca* by high-resolution liquid chromatography and mass spectrometry in – electron spray ionization mode.

S. no.	Name of the compound	Ret. time	Mass	Molecular formula	*m/z*	Difference (ppm)
1	2-(Methylthiomethyl)-3-phenyl-2-propenal	1.089	192.0611	C_11_H_12_OS	191.0536	–0.98
2	l-Arabinose	1.141	150.0509	C_5_H_10_O5	149.0437	13.05
3	Allamandin	1.15	308.0883	C_15_H_16_O7	307.081	4.18
4	Diminazene	4.728	281.1388	C_14_H_15_N_7_	340.1524	0.47
5	Poncirin	5.668	594.1895	C_28_H_34_O_14_	653.2034	8.97
6	Forsythiaside	5.806	624.2003	C_29_H_36_O_15_	623.1927	8.18
7	17-beta-(Acetylthio)estra-1,3,5(10)-trien-3-ol acetate	6.282	372.1757	C_22_H_28_O_3_S	371.1683	0.6
8	Isorhamnetin 3-*O*-[b-d-glucopyranosyl-(1->2)-a-l-rhamnopyranoside]	7.118	624.1642	C_28_H_32_O_16_	623.1568	7.77
9	Albanol A	7.577	562.1641	C_34_H_26_O_8_	607.1625	–2.41
10	5′-Butyrylphosphoinosine	7.616	418.0865	C_14_H_19_N_4_O_9_P	477.1001	5.99
11	Salviaflaside methyl ester	12.952	536.1545	C_25_H_28_O_13_	535.1496	–2.88
12	Doripenem	15.103	420.5109	C_15_H_24_N_4_O_6_S_2_	419.1024	9.38
13	*cis*-Resveratrol 4′-*O*-glucuronide	15.927	404.113	C_20_H_20_O_9_	403.1074	–5.67
14	Methyl (9*Z*)-10′-oxo-6,10′-diapo-6-carotenoate	17.636	312.1723	C_20_H_24_O_3_	311.1665	0.86
15	Broussonin C	18.023	312.173	C_20_H_24_O_3_	311.1659	–1.42

### Acute toxicity study

The acute toxicity study determines the therapeutic safety of the pharmacologically active molecule and provides an insight into the lethal dose for the study animal model (Rajput and Khan 2017). This study demonstrated that EEMC was safe up to the dose of 2000 mg/kg body weight. Behaviour of the animals was evaluated continuously for 3 h and then at an interval of 4 h up to 48 h. Although mild behavioural changes were observed, the treatment did not cause mortality in any mice up to the concentration of 2000 mg/kg. The results of the LD_50_ study performed on mice were expressed using Karber’s method.

### Anxiolytic activity

#### Elevated plus maze test

The anxiolytic effects of EEMC in comparison with that of diazepam as observed in the elevated plus maze test are given in Table 3. The study showed that the number of entries and time spent in open arms exhibited by EEMC treated group (6.25 ± 1.11 and 184.45 ± 1.00, respectively) were remarkably greater in comparison with that of the results seen in closed arm at both the doses. Yet, no significant change in the number of entries in closed arm was seen in case of 100 mg/kg treatment group. Incidentally, diazepam treatment at 1 mg/kg (6.10 ± 0.05 and 180.02 ± 0.40) exhibited similar effects. The findings suggest that the anxiolytic activity exerted by EEMC could be comparable with that of the standard drug.

**Table 3.** Anxiolytic effects of *M. champaca* leaves extract and diazepam in elevated plus maze test.

Groups	Open arms		Closed arms	
	Number of entries	Time spent	Number of entries	Time spent
Control	3.05 ± 0.31^a^	120.04 ± 0.64^a^	5.00 ± 1.20^b^	166.05 ± 0.56^c^
100 mg/kg	4.15 ± 0.76^b^	150.78 ± 2.02^b^	4.78 ± 1.05^b^	127.48 ± 1.04^b^
200 mg/kg	6.25 ± 1.11^c^	184.45 ± 1.00^c^	3.78 ± 0.13^a^	111.09 ± 0.99^a^
Diazepam 1 mg/kg	6.10 ± 0.05^c^	180.02 ± 0.40^c^	4.85 ± 3.01^b^	125.07 ± 0.88^b^

Values are expressed as mean ± SE. Means in the same column with distinct superscripts are significantly different (*p* ≤ 0.05) as separated by Duncan's multiple range test (the different alphabets indicate the statistically significant difference between the values).

#### Light and dark test

As recorded in Table 4, the results of the present study demonstrate notable anxiolytic effects of EEMC assessed using light and dark boxes. The study revealed that there was a significant increase in the time spent in light cubicle at both the doses. However, the treatment using 200 mg/kg body weight (46.86 ± 0.03%) fared optimal among the two doses tested and was better than that exhibited by the standard drug diazepam (44.33 ± 0.64%).

**Table 4.** Anxiolytic effects of *M. champaca* leaves extract and diazepam based on percentage of time spent in light cubicle.

Groups	% time spent in light cubicle
Control	25.08 ± 1.91^a^
100 mg/kg	42.15 ± 0.54^b^
200 mg/kg	46.86 ± 0.03^b^
Diazepam 1 mg/kg	44.33 ± 0.64^b^

Values are expressed as mean ± SE. Means in the same column with distinct superscripts are significantly different (*p* ≤ 0.05) as separated by Duncan's multiple range test (the different alphabets indicate the statistically significant difference between the values).

#### Anti-depression activity

##### Forced swim test

There was no significant difference in the duration of immobility among the different groups tested. A significant delay in the onset of immobility was observed in the EEMC (100 and 200 mg/kg body weight) treated groups along with significantly lowered the immobility time after seven days of treatment. In comparison with the control group which was 146.5 s, the EEMC treated groups at both the doses (93.7 ± 1.70 and 89.1 ± 0.40) fared significantly better and was comparable with that of the standard drug (72.7 ± 3.72) as shown in Table 5.

**Table 5.** Effects of EEMC on immobility time in mouse using forced swim test (FST).

Groups	Treatment	Immobility time (s)	
		1st day	7th day
I	Control group treated with normal saline (10 mL/kg)	154.51 ± 3.87^d^	146.55 ± 3.73^c^
II	Standard drug imipramine (15 mg/kg)	96.90 ± 9.02^a^	72.70 ± 3.72^a^
III	Ethanol extract *M. champaca* leaves (EEMC-100 mg/kg)	144.25 ± 0.94^c^	93.77 ± 1.70^b^
IV	Ethanol extract *M. champaca* leaves (EEMC-200 mg/kg)	134.20 ± 1.07^b^	89.12 ± 0.40^b^

Values are expressed as mean ± SE. Means in the same column with distinct superscripts are significantly different (*p* ≤ 0.05) as separated by Duncan's multiple range test (the different alphabets indicate the statistically significant difference between the values).

##### Tail suspension test

In TST, on day 1, there were no significant differences in the durations of immobility among the different groups. On day 7, all animals of the test groups showed significant improvement in their response to depression. The EEMC extract (100 and 200 mg/kg body weight) treated groups exhibited significant delay in the onset of immobility and significantly reduced the time of immobility in the TST after seven days treatment, which was statistically significant and comparable with that of the control group in which the immobility time was 81.06 s. Comparison between groups I, II, III and IV was made and there was a significant reduction in the immobility time in comparison with that of the control (81.06 ± 3.24 s). However, the results of the standard drugs (35.05 ± 2.75 s) were significantly better (38.50 ± 4.10 and 30.01 ± 2.00 s) for 100 and 200 mg/kg body, respectively on day 7 as shown in Table 6.

**Table 6.** Effects of EEMC on immobility time in mouse using tail suspension test (TST).

Groups	Treatment	Immobility time (s)	
		1st day	7th day
I	Control group treated with normal saline (10 mL/kg)	75.54 ± 1.55^b^	81.06 ± 3.24^c^
II	Standard drug imipramine (15 mg/kg)	45.87 ± 2.30^b^	35.05 ± 2.75^b^
III	Ethanol extract *M. champaca* leaves (EEMC-100 mg/kg)	43.36 ± 1.99^b^	38.50 ± 4.10^b^
IV	Ethanol extract *M. champaca* leaves (EEMC-200 mg/kg)	36.00 ± 5.07^a^	30.01 ± 2.00^a^

Values are expressed as mean ± SE. Means in the same column with distinct superscripts are significantly different (*p* ≤ 0.05) as separated by Duncan's multiple range test (the different alphabets indicate the statistically significant difference between the values).

### Molecular docking and virtual screening

The virtual screening of phytoconstituents based on the criteria of their binding affinity, total non-bonding interactions and hydrogen bonds are given in Tables 7 and 8 for human potassium channel KCSA-FAB (PDB ID: 4UUJ) and human serotonin transporter (PDB ID: 516X), respectively. In this investigation, the human potassium channel KCSA-FAB and human serotonin transporter proteins were used to screen anxiolytic and antidepressant docking analysis, respectively. Out of the 69 compounds, nine compounds were selected based on the above-mentioned criteria. Further, these compounds were selected for drug-likeness and toxicity analysis to select the most potent drug candidate. Diazepam was used as a reference drug for the *in silico* inhibition of human potassium channel KCSA-FAB, whereas imipramine was used against human serotonin transporter as the same.

**Table 7.** Binding affinity, total number of non-bonding interactions, and hydrogen bonds formed by phytoconstituents of *M. champaca* with human potassium channel KCSA-FAB (PDB ID: 4UUJ).

S. no.	Name of the compound	Binding affinity (kJ/mol)	Total no. non-bonding interactions	Total no. of hydrogen bonds
1	(9*Z*,11*E*,13*E*,15*Z*)-4-Oxo-9,11,13,15-octadecatetraenoic acid	–5.9	10	4
2	(*E*,*E*)-1,7-Diphenyl-4,6-heptadien-3-ol	–6.2	5	1
3	1-(beta-d-Ribofuranosyl)-1,4-dihydronicotinamide	–6.6	7	6
4	1-Dehydro-9-fluoro-11-oxotestololactone	–8.8	6	5
**5**	**1-Octen-3-yl glucoside**	**–6.5**	**11**	**7**
**6**	**1-Phosphatidyl-1D-myoinositol 3-phosphate**	**–7.2**	**10**	**10**
7	2-(Methylthiomethyl)-3-phenylpropenal	–5.0	4	2
8	3,6-Dimethoxyestra-1,3,5(10),6,8-pentaene-17-beta-carboxylic acid methyl ester	–7.8	9	5
9	3-Methyldioxyindole	–6.0	4	2
10	3-Phenylpropyl propionate	–5.1	2	0
**11**	**5′-Butyrylphosphoinosine**	**–9.8**	**11**	**6**
12	6-(1,2,3,4-Tetrahydro-6-methoxy-2-naphthyl)-2(1H)-pyridone	–7.4	3	1
13	17-beta-(Acetylthio)estra-1,3,5(10)-trien-3-ol acetate	–8.1	7	1
14	7-*O*-Acetylaustroinulin	–10.1	1	0
15	17-Hydroxylinolenic acid	–5.5	8	2
16	19-Noretiocholanolone	–7.2	2	0
17	Alangimarckine	–8.8	6	1
18	Albanol A	–7.4	7	2
19	Allamandin	–7.6	6	4
20	Austinol	–8.6	1	1
21	Azafrin	–7.4	4	2
22	Beclomethasone (2)	–7.9	8	7
23	Betaxolol	–5.5	9	4
24	Broussonin C	–7.7	4	1
25	Butyl 2-aminobenzoate	–5.4	6	4
26	Carbosulfan	–6.0	5	1
27	*trans*-1,4-*bis*(2-Chlorobenzaminomethyl)cyclohexane	–7.8	4	3
28	*cis*-Resveratrol 4′-*O*-glucuronide	–8.2	5	3
29	Deoxytubulosine	–8.8	6	1
**30**	**Dihydrodeoxystreptomycin**	**–8.2**	**15**	**9**
31	Dihydrospheroidene	–5.2	3	0
32	Diminazene	–7.7	7	3
33	Diplodiatoxin	–6.7	8	3
**34**	**Doripenem**	**–9.2**	**9**	**9**
35	Exemestane	–7.8	0	0
36	Fluticasone propionate	–7.7	3	2
**37**	**Forsythiaside**	**–8.6**	**14**	**8**
38	Harderoporphyrin	–8.3	8	1
39	Heteratisine	–7.4	4	1
40	C-16 Sphinganine	–5.5	6	0
41	Hydroflumethiazide	–6.9	7	5
42	Hydroxyprogesterone caproate	–6.7	1	1
43	Imazamethabenz	–6.2	6	4
**44**	**Isorhamnetin 3-*O*-[b-**d-**glucopyranosyl-(1-2)-a-**l-**rhamnopyranoside]**	**–11.2**	**8**	**7**
45	Kanamycin	–7.6	10	9
46	l-Arabinose	–5.1	2	2
47	Lentiginosine	–5.5	7	3
48	Linoleoylethanolamide	–5.5	6	3
49	Longistylin C	–7.1	5	2
50	Lyngbyatoxin	–7.6	5	0
51	Memantine	–5.2	2	2
52	Methyl (9*Z*)-10′-oxo-6,10′-diapo-6-carotenoate	–5.0	3	1
53	Methyldopa	–6.1	5	4
54	MG(226(4*Z*_7*Z*_10*Z*_13*Z*_16*Z*_1 9*Z*)0000)	–7.1	5	3
55	Mitoxantrone	–7.7	9	6
56	Momilactone A	–7.9	3	1
57	Norethindrone enanthate	–7.1	2	1
58	Panaquinquecol 1	–5.5	7	1
59	Pheophorbide b	–8.9	6	1
60	Pheophorbide a	–9.2	12	4
**61**	**Poncirin**	**–9.6**	**8**	**6**
62	Pyropheophorbide a	–9.1	5	2
**63**	**Salviaflaside methyl ester**	**–7.7**	**10**	**7**
64	Sodium glycocholate	–8.3	4	4
65	Sphinganine	–5.6	3	2
66	Trenbolone	–7.4	5	1
67	Veraguensin	–7.3	6	1
68	Virol B	–5.3	5	0
69	Vulgarone A	–6.3	3	1
70	Diazepam	–6.7	6	3

The compound names and their docking results given in bold indicate the potential hit compounds from docking simulation.

**Table 8.** Binding affinity, total number of non-bonding interactions, and hydrogen bonds formed by phytoconstituents of *M. champaca* with human serotonin transporter (PDB ID: 516X).

S. no.	Name of the compound	Binding affinity (kJ/mol)	Total no. of non-bonding interactions	Total no. of hydrogen bonds
1	(9*Z*,11*E*,13*E*,15*Z*)-4-Oxo-9,11,13,15-octadecatetraenoic acid	–7.1	11	1
2	(*E*,*E*)-1,7-Diphenyl-4,6-heptadien-3-ol	–8.8	9	0
3	1-(beta-d-Ribofuranosyl)-1,4-dihydronicotinamide	–7.5	7	3
4	1-Dehydro-9-fluoro-11-oxotestololactone	–10.0	3	2
**5**	**1-Octen-3-yl glucoside**	**–7.8**	**9**	**6**
**6**	**1-Phosphatidyl-1D-myoinositol-3-phosphate**	**–7.6**	**14**	**7**
7	2-(Methylthiomethyl)-3-phenylpropenal	–6.3	9	0
8	3,6-Dimethoxyestra-1,3,5(10),6,8-pentaene-17-beta-carboxylic acid methyl ester	–8.7	9	2
9	3-Methyldioxyindole	–6.7	6	5
10	3-Phenylpropyl propionate	–6.7	7	0
**11**	**5′-Butyrylphosphoinosine**	**–10.4**	**17**	**4**
12	6-(1,2,3,4-Tetrahydro-6-methoxy-2-naphthyl)-2(1H)-pyridone	–9.4	9	1
13	17-beta-(Acetylthio)estra-1,3,5(10)-trien-3-ol acetate	–9.2	5	0
14	7-*O*-Acetylaustroinulin	–12.9	7	0
15	17-Hydroxylinolenic acid	–6.9	10	1
16	19-Noretiocholanolone	–9.3	6	1
17	Alangimarckine	–11.3	11	0
18	Albanol A	–9.7	5	0
19	Allamandin	–8.3	1	0
20	Austinol	–10.1	5	2
21	Azafrin	–9.0	9	2
22	Beclomethasone (2)	–8.4	3	2
23	Betaxolol	–7.1	10	2
24	Broussonin C	–9.6	13	1
25	Butyl 2-aminobenzoate	–6.7	10	2
26	Carbosulfan	–7.9	7	1
27	*trans*-1,4-*bis*(2-Chlorobenzaminomethyl)cyclohexane	–10.3	5	3
28	*cis*-Resveratrol 4′-*O*-glucuronide	–9.2	5	3
29	Deoxytubulosine	–11.3	12	0
**30**	**Dihydrodeoxystreptomycin**	**–8.3**	**6**	**5**
31	Dihydrospheroidene	–8.6	3	0
32	Diminazene	–8.5	9	4
33	Diplodiatoxin	–8.2	8	3
**34**	**Doripenem**	**–9.8**	**13**	**11**
35	Exemestane	–9.8	1	1
36	Fluticasone propionate	–9.6	8	5
**37**	**Forsythiaside**	**–10.8**	**13**	**6**
38	Harderoporphyrin	–4.9	9	2
39	Heteratisine	–9.0	3	0
40	C-16 Sphinganine	–6.1	8	1
41	Hydroflumethiazide	–7.9	8	2
42	Hydroxyprogesterone caproate	–9.6	5	0
43	Imazamethabenz	–8.8	4	0
**44**	**Isorhamnetin 3-*O*-[b-**d-**glucopyranosyl-(1-2)-a-**l-**rhamnopyranoside]**	**–12.5**	**11**	**7**
45	Kanamycin	–7.9	6	5
46	l-Arabinose	–5.2	5	3
47	Lentiginosine	–6.0	3	1
48	Linoleoylethanolamide	–7.1	10	2
49	Longistylin C	–9.4	11	1
50	Lyngbyatoxin	–9.6	8	0
51	Memantine	–7.0	3	1
52	Methyl (9*Z*)-10′-oxo-6,10′-diapo-6-carotenoate	–7.1	5	1
53	Methyldopa	–6.7	5	2
54	MG(226(4*Z*_7*Z*_10*Z*_13*Z*_16*Z*_1 9*Z*)0000)	–7.0	5	1
55	Mitoxantrone	–7.7	16	3
56	Momilactone A	–9.7	3	1
57	Norethindrone enanthate	–10.2	10	2
58	Panaquinquecol 1	–7.7	10	0
59	Pheophorbide b	–4.3	12	1
60	Pheophorbide a	–4.9	14	2
**61**	**Poncirin**	**–11.3**	**15**	**7**
62	Pyropheophorbide a	–6.3	9	0
**63**	**Salviaflaside methyl ester**	**–9.8**	**12**	**7**
64	Sodium glycocholate	–9.0	3	1
65	Sphinganine	–7.3	8	3
66	Trenbolone	–10.3	9	2
67	Veraguensin	–8.3	13	2
68	Virol B	–7.5	9	2
69	Vulgarone A	–8.6	5	0
70	Imipramine	–8.5	6	0

The compound names and their docking results given in bold indicate the potential hit compounds from docking simulation.

### Drug-likeness and toxicity analysis

OSIRIS property explorer uses Lipinski’s rule of five for the analysis of drug-likeness and toxicity of the compounds. Lipinski’s rule of five states that a potential drug candidate must have its molecular weight ≤500 Da, cLog*P* value (logarithm of its partition coefficient between *n*-octanol and water log(*c*_octanol_/*c*_water_) ≤4.15, hydrogen bond acceptors ≤10, hydrogen bond donors ≤5 and rotatable bonds ≤10) (Lipinski et al. 2001). Based on these parameters, nine compounds obtained from previous virtual screening were assessed for their drug-likeness and toxicity. Out of the nine compounds, only doripenem was predicted with no violation of Lipinski’s rule of five. It was also predicted with no risk of causing mutagenic, tumorigenic, irritating and reproductive aberrations. Although reference drugs diazepam and imipramine showed no violations of Lipinski’s rule of five, their drug scores were found to be lower than doripenem. Besides, during the toxicity analysis, diazepam was predicted with a high risk showing possible mutagenicity, tumorigenicity and reproductive health aberrations. In addition, imipramine was also predicted with reproductive health aberrations. Apart from docking simulations, drug-likeness and toxicity studies also favour doripenem in comparison with the reference drugs (Table 9).

**Table 9.** ADMET profile of the *M. champaca* phytoconstituents and standard drugs.

Compounds	Drug-likeness parameters						Toxicological parameters			
	cLog*P*	Mol. weight (g/mol)	HBA	HBD	nRB	Drug- score	Mutagenicity	Tumorigenicity	Irritability	Reproductive effectivity
1-Octen-3-yl glucoside	0.66	290.35	6	4	8	0.23	No risk	High risk	Medium risk	No risk
1-Phosphatidyl-1D-myoinositol 3-phosphate	–9.13	470.21	16	9	10	0.40	No risk	No risk	No risk	No risk
5′-Butyrylphosphoinosine	–3.28	418.30	13	4	8	0.43	No risk	No risk	No risk	No risk
Dihydrodeoxystreptomycin	–7.65	583.59	19	13	9	0.62	No risk	No risk	No risk	No risk
**Doripenem**	**–3.68**	**420.51**	**10**	**5**	**6**	**0.86**	**No risk**	**No risk**	**No risk**	**No risk**
Forsythiaside	–0.38	624.59	15	9	11	0.34	No risk	No risk	No risk	No risk
Isorhamnetin 3-*O*-[b-d-glucopyranosyl-(1-2)-a-l-rhamnopyranoside]	–0.98	624.55	16	9	7	0.32	No risk	No risk	No risk	No risk
Poncirin	–0.47	594.56	14	7	7	0.43	No risk	No risk	No risk	No risk
Salviaflaside methyl ester	–0.11	536.48	13	7	11	0.35	No risk	No risk	No risk	No risk
**Diazepam**	**2.98**	**–4.67**	**3**	**0**	**1**	**0.20**	**High risk**	**High risk**	**No risk**	**High risk**
**Imipramine**	**3.89**	**280.41**	**2**	**0**	**4**	**0.51**	**No risk**	**No risk**	**No risk**	**High risk**

cLog*P*: logarithm of its partition coefficient between n-octanol and water log(*c*_octanol_/*c*_water_); HBA: hydrogen bond acceptors; HBD: hydrogen bond donors; nRB: number of rotatable bonds.

Bold values indicate the result of selected compounds which were taken for ADMET studies.

### Interaction analysis of doripenem with target proteins

**Figure 1.**
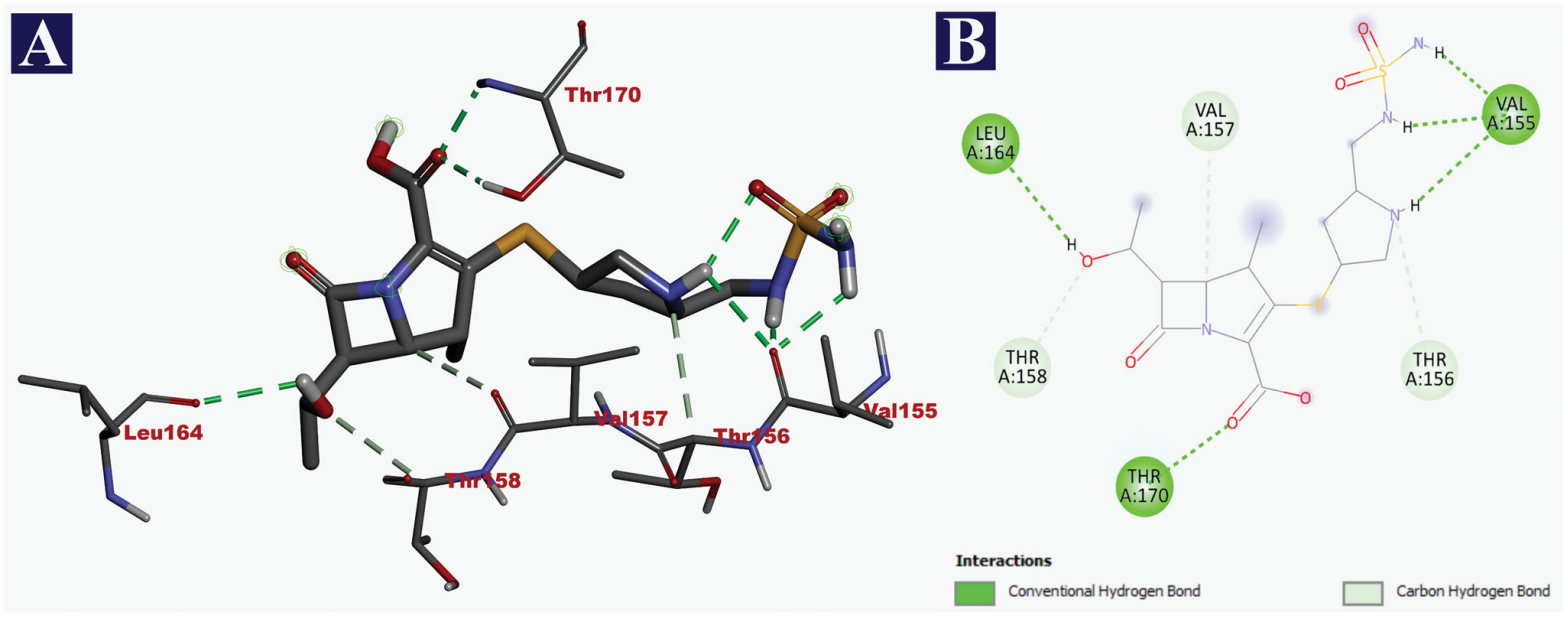
Visualization of docking analysis of doripenem binding with human potassium channel KCSA-FAB (PDB ID: 4UUJ). (A) 3D representation. (B) 2D representation.

**Figure 2.**
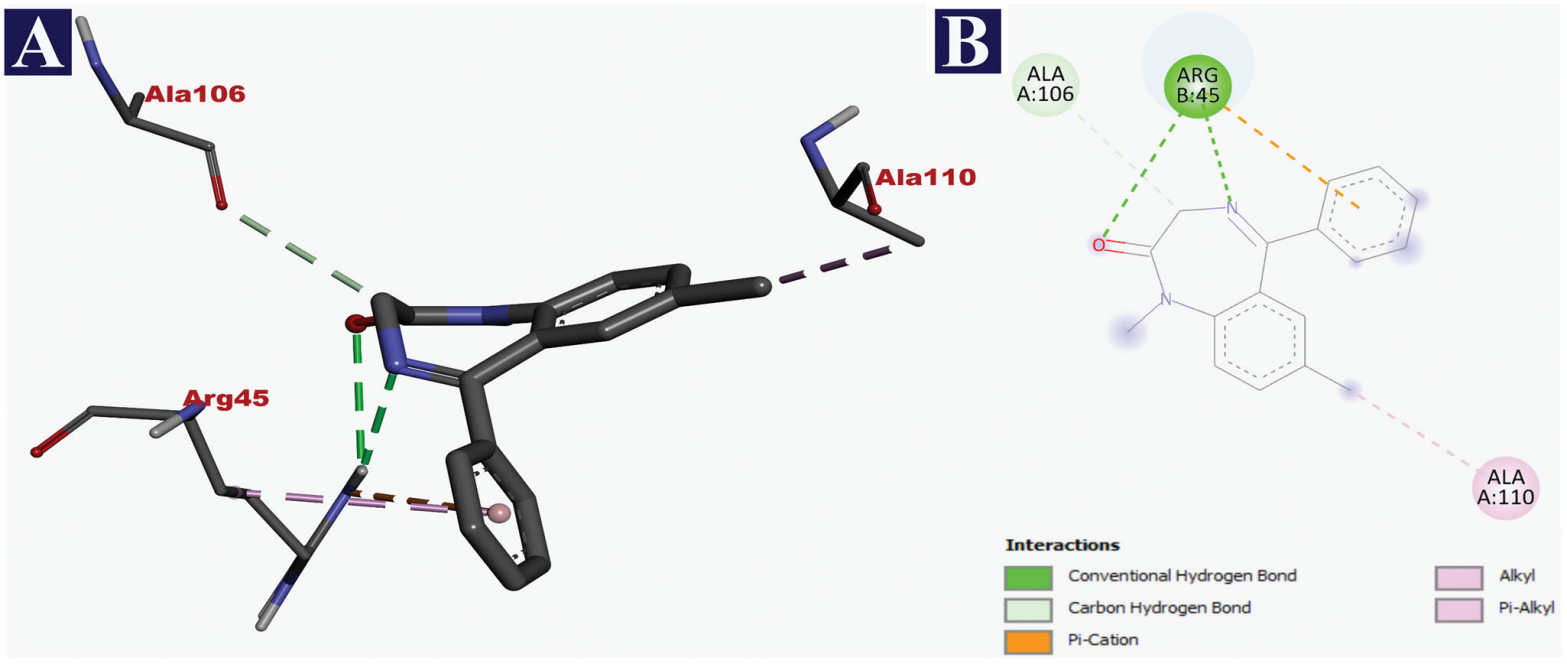
Visualization of docking analysis of diazepam binding with human potassium channel KCSA-FAB (PDB ID: 4UUJ). (A) 3D representation. (B) 2D representation.

Doripenem was bound with the fragment antigen-binding (FAB) site of the human potassium channel KCSA-FAB. The phytochemical binds to the external surface of the protein within the hydrophobic binding pocket. The FAB domain remains outside the cell to facilitate the binding of the antigens. Doripenem binds through nine non-bonding interactions, all of them being hydrogen bonds with a binding affinity of −9.2 kJ/mol. It formed hydrogen bonds with THR 170 (2.16 Å), THR 170 (2.25 Å), LEU 164 (2.61 Å), VAL 155 (2.65 Å), VAL 155 (1.83 Å), VAL 155 (2.32 Å), THR 156 (3.62 Å), THR 158 (3.53 Å) and VAL 157 (3.52 Å) (Table 10). The binding interactions of doripenem with human potassium channel KCSA-FAB are given in Figure 1. Binding at this site is expected to reduce the influx of potassium ions, which reduces the neuronal excitability, ultimately resulting in a relaxed state of mind leading to the expected anxiolytic activity. In comparison with doripenem, the reference drug diazepam could form only six non-bonding interactions with a binding affinity of −6.7 kJ/mol. Diazepam formed only three hydrogen bonds with ARG 45 (2.50 Å), ARG 45 (3.05 Å) and ALA 106 (3.49 Å). It also formed a lone electrostatic bond with ARG 45 (4.40 Å), and two hydrophobic/interactions (pi-alkyl and alkyl) with ARG 45 (4.24 Å) and ALA 110 (3.74 Å), respectively (Table 10). The binding interactions of diazepam with the human potassium channel KCSA-FAB are depicted in Figure 2.

**Table 10.** Binding interactions of doripenem and diazepam with human potassium channel (PDB ID: 4UUJ) along with their respective bond lengths (Å).

Name of the compound	Binding affinity (kJ/mol)	Hydrogen bonds	Electrostatic bonds	Hydrophobic bonds	
				Pi-Alkyl	Alkyl
Doripenem	–9.2	THR 170 (2.16), THR 170 (2.25), LEU 164 (2.61), VAL 155 (2.65), VAL 155 (1.83), VAL 155 (2.32), THR 156 (3.62), THR 158 (3.53), VAL 157 (3.52)	–	–	–
Diazepam	–6.7	ARG 45 (2.50), ARG 45 (3.05), ALA 106 (3.49)	ARG 45 (4.40)	ARG 45 (4.24)	ALA 110 (3.74)

**Figure 3.**
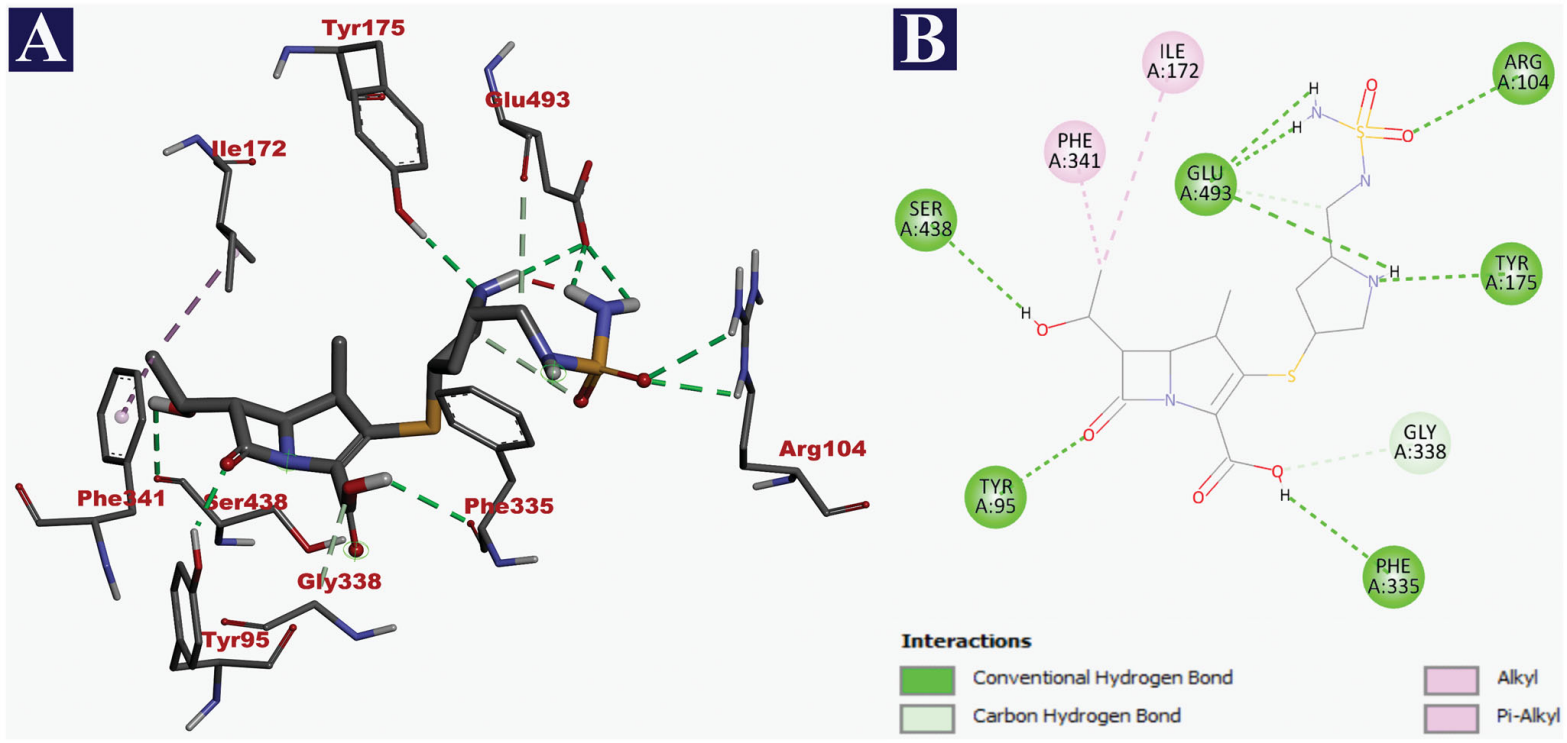
Visualization of docking analysis of doripenem binding with human serotonin transporter (PDB ID: 516X). (A) 3D representation. (B) 2D representation.

**Figure 4.**
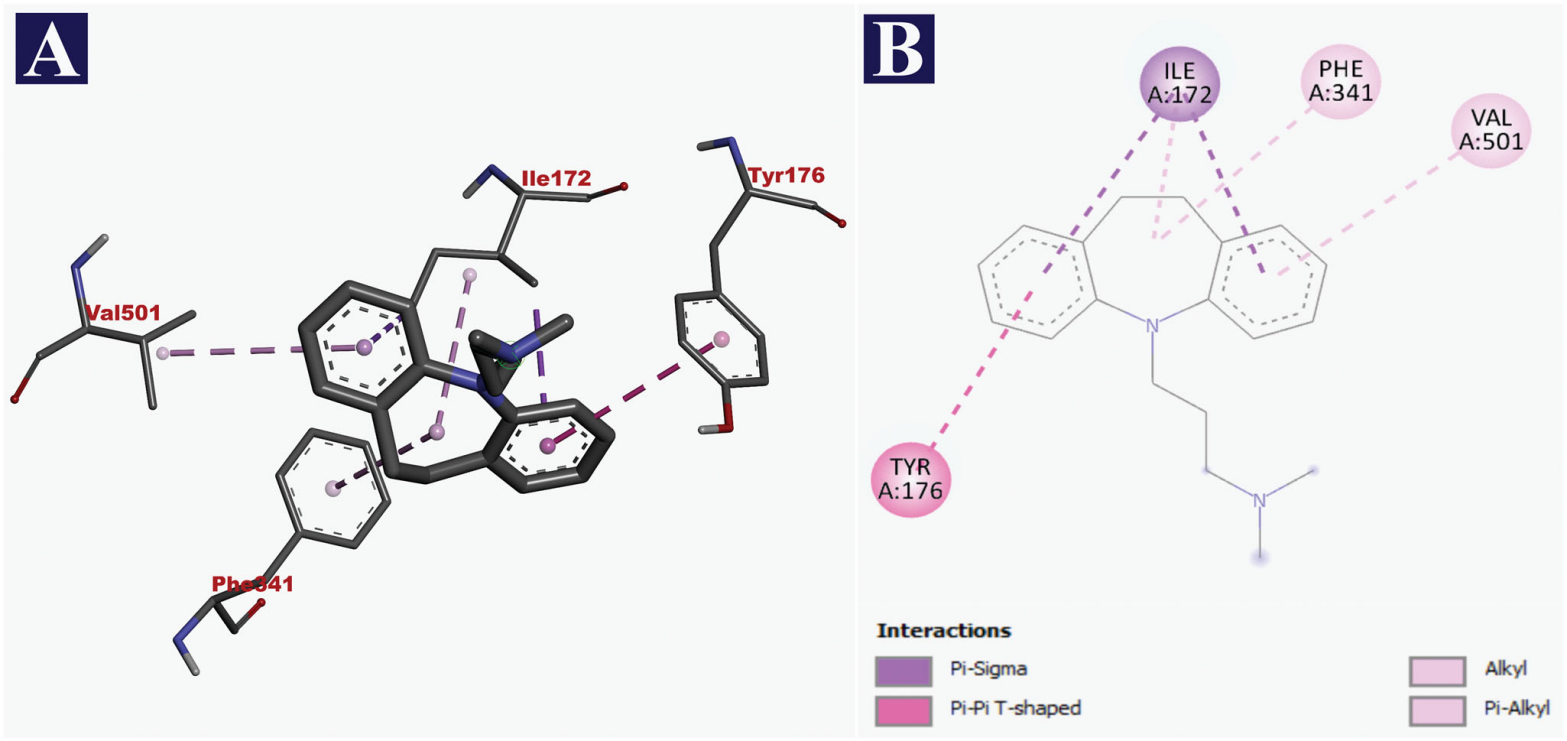
Visualization of docking analysis of imipramine binding with human serotonin transporter (PDB ID: 516X). (A) 3D representation. (B) 2D representation.

In case of human serotonin transporter, doripenem was bound to the central binding site of the hydrophobic binding pocket, in the exact site of the co-crystal ligand paroxetine with a binding affinity of −9.8 kJ/mol. The hydrophobic binding pocket was created by 10 transmembrane helices (TM) of the protein. Out of 13 non-bonding interactions, doripenem formed 11 hydrogen bonds with SER 438 (2.71 Å), PHE 335 (2.63 Å), GLU 493 (2.87 Å), GLU 493 (2.71 Å), GLU 493 (2.79 Å), TYR 95 (2.02 Å), ARG 104 (2.55 Å), ARG 104 (2.75 Å), TYR 175 (2.46 Å), GLU 493 (3.63 Å) and GLY 338 (3.69 Å). It also formed two hydrophobic interactions (pi-alkyl and alkyl) with PHE 341 (4.59 Å) and ILE 172 (3.89 Å), respectively (Table 11). The binding interactions of doripenem are given in Figure 3. In comparison with doripenem, imipramine reference drug could bind with the binding affinity of −8.5 kJ/mol. It formed a total of six non-bonding interactions, with all of them being hydrophobic bonds. It bound to the central binding site of the hydrophobic binding pocket. In the context, two pi-sigma bonds with ILE 172 (3.90 Å) and ILE 172 (3.62 Å), a single pi–pi interaction with TYR 176 (5.04 Å), two pi-alkyl bonds with PHE 341 (4.63 Å) and VAL 501 (5.00 Å), and another single alkyl bond with ILE 172 (4.56 Å) were formed by imipramine (Table 11). The details of the interaction of imipramine with human serotonin transporter are depicted in Figure 4.

**Table 11.** Binding interactions of doripenem and imipramine with human serotonin transporter (PDB ID: 516X) along with their respective bond lengths (Å).

Name of the compound	Binding affinity (kJ/mol)	Hydrogen bonds	Hydrophobic bonds			
			Pi–sigma	Pi–Pi	Pi–alkyl	Alkyl
Doripenem	–9.8	SER 438 (2.71), PHE 335 (2.63), GLU 493 (2.87), GLU 493 (2.71), GLU 493 (2.79), TYR 95 (2.02), ARG 104 (2.55), ARG 104 (2.75), TYR 175 (2.46), GLU 493 (3.63), GLY 338 (3.69)	–	–	PHE 341 (4.59)	ILE 172 (3.89)
Imipramine	–8.5	–	ILE 172 (3.90), ILE 172 (3.62)	TYR 176 (5.04)	PHE 341 (4.63), VAL 501 (5.00)	ILE 172 (4.56)

## Discussion

Anxiety and depression are mental conditions considered among the most studied psychiatric diseases in humans. About 1/8th of the world population suffers from these conditions and at least 10% of the people suffer from it once in their entire life (Porter and Meldrum 2009). Studies have proven the beneficiary effects of flavonoids and other plant phytochemicals that have been efficacious in treating mild to moderate conditions (Gerzson et al. 2012). However, the exact mechanism of action of these drugs is less exploited and hence they cannot be established as commercial drugs for the treatment of depression and anxiety.

In our study, preliminary screening of the various phytochemicals present in the ethanol extract of *M. champaca* leaves was performed. It was revealed that the extract was abundant in alkaloids, tannins, steroids, phenols, flavonoids, carbohydrates and glycosides. Previous study evaluated the free radical scavenging activity of the flower extract that demonstrated compounds such as anonaine, an aporphine (isoquinoline) alkaloids present in the extract showed significant pharmacological activities such as, antibacterial, antifungal, antioxidative, anticancer and antidepressant (Li et al. 2013).

The study also evaluated the toxicity of the extract that revealed the extract was not toxic up to a dose of 2000 mg/kg body administration. In this study, the anxiolytic activity of the extract was assessed using two standard assays namely, elevated plus maze and light and dark tests. In comparison with the results of the standard drug diazepam, 100 and 200 mg/kg body weight treatment of the extract demonstrated significant activity in lowering anxiety. The number of entries and time spent in open arm increased remarkably with a corresponding decrease in the number of entries and time spent in the closed arms. Mice experience anxiety because of the fear of height when placed in the elevated plus maze design. This is represented in the motor activity and preferences shown by the mice to stay in its safe zone (Walia et al. 2018). Agents such as diazepam have proven ability to improve the motor activity in terms of the time spent in the open arms by the experimental animal (Shimizu et al. 2018). Previous study on the anxiolytic activity of *Nymphaea alba* L. (Nymphaeaceae) revealed a significant enhancement in time spent and number of entries in the open arm and a decrease in the duration of immobility in light box (Thippeswamy et al. 2011). The results from the present study are in agreement with this study revealing that the ability of the extract to lower anxiety induced by height in mice, which is on par with that of diazepam, the standard drug. The light and dark assay is yet another test to evaluate the anxiolytic activity of drug molecules. The increased percentage of time spent in the light compartment indicates improved anxiolytic activity. In our study, the anxiolytic activity exerted by the extract was significant in comparison with that of the standard drug diazepam at both the doses with extract treated at 200 mg/kg concentration was better than 100 mg/kg concentration. In yet another study, *Cynodon dactylon* (L.) Pers. (Poaceae) extract on albino mice demonstrated remarkable antidepressant and anxiolytic potential, which was indicated to be owing to the presence of high flavonoid content in the extract (Kothari 2021). Flavones present in the plant extracts are known to bind to the benzodiazepine site of the GABA adrenergic receptor that is expected to bring about the observed anxiolytic effects. Several plant flavones have been assessed based on their traditional uses to reduce anxiety (Liu et al. 2021). In a study by Malathi and Ravindran (2015), the antidepressant and anxiolytic activities of the methanolic extract of its flower were evaluated. It was observed that the extract was potential in reducing anxiety induced by noise by the stimulation of dopamine. Therefore, it can be suggested that the targeted regulation of anxiety by lowering dopamine-mediated signal transduction leads to the observed effects in the present study.

Furthermore, the present study evaluated the antidepressant activity of the extract using FST and TST. In both the tests, immobility shown by the experimental mice on the 1st and 7th day was compared. The results demonstrated that there was more reduction in the immobility on the 7th day than on the 1st day. These results indicate that the EEMC on chronic administration of higher doses has antidepressant effect similar to that of the standard drug imipramine. The two are the established methods for the assessment of depression among various animal models (Shoeb et al. 2013). In humans, a sense of hopelessness is seen that is in turn represented as the paradigm of helplessness as observed by the failure to work towards survival in rodents. The behaviour of immobility after only a short struggle is represented as a depressive behaviour in case of mice that can be extrapolated to the human depressive behaviour (Jahani et al. 2019). In our study, the improved time taken to reach immobility state shows that the extract is indeed beneficiary in bringing about antidepressant activity.

Prediction of ligand–target interactions using *in silico* approaches is gaining momentum in the research of natural products and their bioactivity. There is an added advantage of an insight on the probable binding mechanisms in the protein binding pocket demonstrating its interaction with the ligand. To validate the findings from the biological studies, the present study also evaluated the molecular interaction of phytochemicals from *M. champaca* with the two target proteins, namely human potassium channel KCSA-FAB and human serotonin transporter.

The passive flow of potassium ions in neurons and other body cells is mediated by the human potassium channel KCSA-FAB. An increased potassium influx causes neuronal excitability, which is thought to be the cause of anxiety disorders (Lenaeus et al. 2014). As a result, inhibiting the human potassium channel KCSA-FAB would result in potassium depletion and anxiolytic effects. Doripenem has been reported to interact with the outer FAB complex, which serves as an antigen binding site in this study. This would close the channel, inhibiting potassium ions from entering the cells. Despite the fact that diazepam was bound to the FAB complex, doripenem was chosen over diazepam because of its superior interaction and binding affinity. In case of drug-likeness and toxicity analyses, diazepam was predicted with probable mutagenic, tumorigenic and reproductive health aberrant properties. Alternatively, doripenem was predicted with no risk of inducing toxic effects. Besides, drug score of doripenem is higher than that of diazepam. Collectively, the superior binding interaction, lower binding affinity, higher drug-score and zero risk of inducing toxicity make doripenem a better potential drug candidate, in comparison with the conventional drug diazepam.

The regulation of serotonin levels is aided by human serotonin transporters. Serotonin regulates the functioning of the central nervous system as well as other bodily functions such as digestion, cardiovascular function and reproduction. Serotonin is released into the synaptic cleft and diffused by serotonin receptors. Re-uptake of serotonin, on the other hand, causes depression. Antidepressant medicines such as prozac, imipramine and paroxetine have been shown to impede serotonin reuptake, resulting in depression-free state (Coleman et al. 2016). As a result, blocking the human serotonin transporter may pave the way for the development of antidepressant drugs. Doripenem occupies the centre of the binding pocket created by all the transmembrane helices during the molecular docking simulation. Despite the fact that imipramine binds to the pocket's core site, its interactions and binding affinity appear to be inferior to doripenem. The blockage of serotonin reuptake would result in a depression-free state. During drug-likeness and toxicity analysis, imipramine was predicted with probable reproductive health aberrations. However, doripenem was found to be predicted with no risk with all the toxicity parameters used. Even the drug-score of doripenem was found to be more than imipramine. Therefore, doripenem could be employed as a strong inhibitor of human serotonin transporter because of the degree of interaction, lower binding affinity, higher drug-likeness and zero risk of probable toxicity, compared to imipramine.

## Conclusions

Based on the biological result interpretations, *M. champaca* leaves extracted using ethanol as a solvent is a rich source of compounds with potential antidepressant and anxiolytic properties. *In vivo* findings also support their traditional importance as potent formulations in the treatment of depression and anxiety. Besides, the molecular docking study revealed that doripenem showed good interaction with both human potassium channel KSCA-FAB and human serotonin transporter proteins docking simulations, in comparison with reference drugs, thereby showing *in silico* anxiolytic and antidepressant activities, respectively. The drug-likeness and toxicity profile of doripenem also favours it as an effective potential drug candidate, whereas reference drugs were reported with toxicity predictions. Overall, this study paves way for future research in understanding the ethnomedicinal uses of *M. champaca* leaves in the treatment of anxiety and depression.

## Data Availability

Data sharing is not applicable to this article as no new data were created or analysed in this study.

## References

[CIT0001] Bhatt S, Nagappa AN, Patil CR. 2020. Role of oxidative stress in depression. Drug Discov Today. 25(7):1270–1276. 32404275 10.1016/j.drudis.2020.05.001

[CIT0002] Chandra K, Salman AS, Mohd A, Sweety R, Ali KN. 2015. Protection against FCA induced oxidative stress induced DNA damage as a model of arthritis and *in vitro* anti-arthritic potential of *Costus speciosus* rhizome extract. Int J Pharmacogn Phytochem Res. 7:383–389.

[CIT0003] Coleman JA, Green EM, Gouaux E. 2016. X-ray structures and mechanism of the human serotonin transporter. Nature. 532(7599):334–339. 27049939 10.1038/nature17629 PMC4898786

[CIT0004] Fauci AS, Kasper DL, Longo DL, Braunwald E, Hauser SL, Jameson JL, Loscalzo J. 2008. Harrison’s principles of internal medicine. New York: McGraw Hill Medical.

[CIT0005] Fedoce ADG, Ferreira F, Bota RG, Bonet-Costa V, Sun PY, Davies KJ. 2018. The role of oxidative stress in anxiety disorder: cause or consequence? Free Radic Res. 52(7):737–750. 29742940 10.1080/10715762.2018.1475733 PMC6218334

[CIT0007] Gerzson MFB, Victoria FN, Radatz CS, de Gomes MG, Boeira SP, Jacob RG, Alves D, Jesse CR, Savegnago L. 2012. *In vitro* antioxidant activity and *in vivo* antidepressant-like effect of α-(phenylselanyl) acetophenone in mice. Pharmacol Biochem Behav. 102(1):21–29. 22484161 10.1016/j.pbb.2012.03.016

[CIT0008] Gong ZH, Li YF, Zhao N, Yang HJ, Su RB, Luo ZP, Li J. 2006. Anxiolytic effect of agmatine in rats and mice. Eur J Pharmacol. 550(1–3):112–116. 17011547 10.1016/j.ejphar.2006.08.057

[CIT0009] Greaney JL, Saunders EF, Santhanam L, Alexander LM. 2019. Oxidative stress contributes to microvascular endothelial dysfunction in men and women with major depressive disorder. Circ Res. 124(4):564–574. 30582458 10.1161/CIRCRESAHA.118.313764 PMC6375800

[CIT0010] Halliwell B, Whiteman M. 2004. Measuring reactive species and oxidative damage *in vivo* and in cell culture: how should you do it and what do the results mean? Br J Pharmacol. 142(2):231–255. 15155533 10.1038/sj.bjp.0705776 PMC1574951

[CIT0011] Hasler G. 2010. Pathophysiology of depression: do we have any solid evidence of interest to clinicians? World Psychiatry. 9(3):155–161. 20975857 10.1002/j.2051-5545.2010.tb00298.x PMC2950973

[CIT0012] Hoffmann JJ, Torrance SJ, Widehopf RM, Cole JR. 1977. Cytotoxic agents from *Michelia champaca* and *Talauma ovata*: parthenolide and costunolide. J Pharm Sci. 66(6):883–884. 889597 10.1002/jps.2600660642

[CIT0013] Hossain MM, Jahangir R, Hasan SR, Akter R, Ahmed T, Islam MI, Faruque A. 2009. Antioxidant, analgesic and cytotoxic activity of *Michelia champaca* Linn. leaf. Stamford. J Pharm Sci. 2(2):1–7.

[CIT0014] Jahani R, Khaledyan D, Jahani A, Jamshidi E, Kamalinejad M, Khoramjouy M, Faizi M. 2019. Evaluation and comparison of the antidepressant-like activity of *Artemisia dracunculus* and *Stachys lavandulifolia* ethanolic extracts: an *in vivo* study. Res Pharm Sci. 14(6):544–553. 32038734 10.4103/1735-5362.272563 PMC6937744

[CIT0015] Jarald EE, Joshi SB, Jain DC. 2008. Antidiabetic activity of flower buds of *Michelia champaca* Linn. Indian J Pharmacol. 40(6):256–260. 21279181 10.4103/0253-7613.45151 PMC3025142

[CIT0016] Jawaid T, Gupta R, Siddiqui ZA. 2011. A review on herbal plants showing antidepressant activity. Int J Pharm Sci Res. 2:3051–3060.

[CIT0017] Khan MR, Kihara M, Omoloso AD. 2002. Antimicrobial activity of *Michelia champaca*. Fitoterapia. 73(7–8):744–748. 12490248 10.1016/s0367-326x(02)00248-4

[CIT0018] Kothari S. 2021. Evaluation of antianxiety and antidepressant activity of aqueous extract of *Cynodon dactylon* (Doob grass) in Swiss albino mice. Int J Green Pharm. 15:182–187.

[CIT0019] Kumar A, Gupta RC, Thomas MA, Alger J, Wyckoff N, Hwang S. 2004. Biophysical changes in normal-appearing white matter and subcortical nuclei in late-life major depression detected using magnetization transfer. Psychiatry Res. 130(2):131–140. 15033183 10.1016/j.pscychresns.2003.12.002

[CIT0020] Kumar V, Ramu R, Shirahatti PS, Kumari VC, Sushma P, Mandal SP, Patil SM. 2021. α-Glucosidase, α-amylase inhibition, kinetics and docking studies of novel (2-chloro-6-(trifluoromethyl) benzyloxy) arylidene) based rhodanine and rhodanine acetic acid derivatives. Chemistry Select. 6(36):9637–9644.

[CIT0021] Lenaeus MJ, Burdette D, Wagner T, Focia PJ, Gross A. 2014. Structures of KcsA in complex with symmetrical quaternary ammonium compounds reveal a hydrophobic binding site. Biochemistry. 53(32):5365–5373. 25093676 10.1021/bi500525s PMC4139162

[CIT0022] Li HT, Wu HM, Chen HL, Liu CM, Chen CY. 2013. The pharmacological activities of (−)-anonaine. Molecules. 18(7):8257–8263. 23857128 10.3390/molecules18078257 PMC6270643

[CIT0023] Lipinski CA, Lombardo F, Dominy BW, Feeney PJ. 2001. Experimental and computational approaches to estimate solubility and permeability in drug discovery and development settings. Adv Drug Deliv Rev. 46(1–3):3–26. 11259830 10.1016/s0169-409x(00)00129-0

[CIT0024] Liu Z, Silva J, Shao AS, Liang J, Wallner M, Shao XM, Li M, Olsen RW. 2021. Flavonoid compounds isolated from Tibetan herbs, binding to GABAA receptor with anxiolytic property. J Ethnopharmacol. 267:113630. 33246118 10.1016/j.jep.2020.113630

[CIT0025] Malathi S, Ravindran R. 2015. Effect of *Michelia champaca* on locomotor activity, anxiety and depression-like behaviours in noise stress induced Wistar albino rats. World J Pharm Res. 4:1409–1426.

[CIT0026] Nickavar B, Kamalinejad M, Haj-Yahya M, Shafaghi B. 2006. Comparison of the free radical scavenging activity of six Iranian *Achillea* species. Pharm Biol. 44(3):208–212.

[CIT0027] Patil SM, Martiz RM, Ramu R, Shirahatti PS, Prakash A, Chandra JS, Ranganatha LV. 2021. *In silico* identification of novel benzophenone-coumarin derivatives as SARS-CoV-2 RNA-dependent RNA polymerase (RdRp) inhibitors. J Biomol Struct Dyn. 11:1–17. 10.1080/07391102.2021.1978322 34632942

[CIT0028] Patil SM, Martiz RM, Ramu R, Shirahatti PS, Prakash A, Kumar BRP, Kumar N. 2021. Evaluation of flavonoids from banana pseudostem and flower (aminoguanidine and catechin) as potent inhibitors of α-glucosidase: an *in silico* perspective. J Biomol Struct Dyn. 6:1–15. 10.1080/07391102.2021.1971561 34488558

[CIT0029] Patil SM, Maruthi KR, Bajpe SN, Vyshali VM, Sushmitha S, Akhila C, Ramu R. 2021. Comparative molecular docking and simulation analysis of molnupiravir and remdesivir with SARS-CoV-2 RNA dependent RNA polymerase (RdRp). Bioinformation. 17(11):932–939. 35655903 10.6026/97320630017932 PMC9148593

[CIT0030] Patil SM, Shirahatti PS, Chandana Kumari VB, Ramu R, Nagendra Prasad MN. 2021. *Azadirachta indica* A. Juss (neem) as a contraceptive: an evidence-based review on its pharmacological efficiency. Phytomedicine. 88:153596. 34092456 10.1016/j.phymed.2021.153596

[CIT0031] Porsolt RD, Bertin A, Jalfre MJ. 1977. Behavioral despair in mice: a primary screening test for antidepressants. Arch Int Pharmacodyn Ther. 229(2):327–336. 596982

[CIT0032] Porter RJ, Meldrum BS. 2009. Antidepressant agents. In: Katzung BG, Masters SB, Trevor AJ, editors. Basic and clinical pharmacology. New Delhi: Tata McGraw Hill; p. 521–525.

[CIT0033] Rajput MA, Khan RA. 2017. Phytochemical screening, acute toxicity, anxiolytic and antidepressant activities of the *Nelumbo nucifera* fruit. Metab Brain Dis. 32(3):743–749. 28144887 10.1007/s11011-017-9963-x

[CIT0034] Reddy MS. 2010. Depression: the disorder and the burden. Indian J Psychol Med. 32(1):1–2. 21799550 10.4103/0253-7176.70510 PMC3137804

[CIT0035] Riaz A, Khan RA. 2014. Effect of *Punica granatum* on behavior in rats. Afr J Pharm Pharmacol. 8:1118–1126.

[CIT0036] Shimizu T, Minami C, Mitani A. 2018. Effect of electrical stimulation of the infralimbic and prelimbic cortices on anxiolytic-like behavior of rats during the elevated plus-maze test, with particular reference to multiunit recording of the behavior-associated neural activity. Behav Brain Res. 353:168–175. 30057351 10.1016/j.bbr.2018.07.005

[CIT0037] Shoeb A, Chowta M, Pallempati G, Rai A, Singh A. 2013. Evaluation of antidepressant activity of vanillin in mice. Indian J Pharmacol. 45(2):141–144. 23716889 10.4103/0253-7613.108292 PMC3660925

[CIT0038] Steru L, Chermat R, Thierry B, Simon P. 1985. The tail suspension test: a new method for screening antidepressants in mice. Psychopharmacology. 85(3):367–370. 3923523 10.1007/BF00428203

[CIT0039] Swantara IMD, Bawa IGAG, Suprapta DN, Agustina KK, Temaja IGRM. 2020. Identification *Michelia alba* barks extract using gas chromatography-mass spectrometry (GC–MS) and its antifungal properties to inhibit microbial growth. Biodiversitas. 21(4):1541–1550.

[CIT0040] Taprial S. 2015. A review on phytochemical and pharmacological properties of *Michelia champaca* Linn. Family: Magnoliaceae. Int J Pharmacogn. 2:430–436.

[CIT0041] Thippeswamy BS, Mishra B, Veerapur VP, Gupta G. 2011. Anxiolytic activity of *Nymphaea alba* Linn. in mice as experimental models of anxiety. Indian J Pharmacol. 43(1):50–55. 21455422 10.4103/0253-7613.75670 PMC3062121

[CIT0042] Trott O, Olson AJ. 2010. AutoDock Vina: improving the speed and accuracy of docking with a new scoring function, efficient optimization, and multithreading. J Comput Chem. 31(2):455–461. 19499576 10.1002/jcc.21334 PMC3041641

[CIT0043] Tsegay A, Damte A, Kiros A. 2020. Determinants of suicidal ideation among patients with mental disorders visiting psychiatry outpatient unit in Mekelle town, psychiatric clinics, Tigray, Northern Ethiopia: a case-control study. Ann Gen Psychiatr. 19(1):1–12. 10.1186/s12991-020-00270-x PMC7066829 32190099

[CIT0044] Walf AA, Frye CA. 2007. The use of the elevated plus maze as an assay of anxiety-related behavior in rodents. Nat Protoc. 2(2):322–328. 17406592 10.1038/nprot.2007.44 PMC3623971

[CIT0045] Walia V, Garg C, Garg M. 2018. Anxiolytic-like effect of pyridoxine in mice by elevated plus maze and light and dark box: evidence for the involvement of GABAergic and NO-sGC-cGMP pathway. Pharmacol Biochem Behav. 173:96–106. 30040985 10.1016/j.pbb.2018.06.001

[CIT0046] Wang HM, Lo WL, Huang LY, Wang YD, Chen CY. 2010. Chemical constituents from the leaves of *Michelia alba*. Nat Prod Res. 24(5):398–406. 20306361 10.1080/14786410802394132

